# Unveiling the aging-immune axis in irritable bowel syndrome: a multi-omics and machine learning approach to biomarker discovery and validation

**DOI:** 10.3389/fimmu.2026.1893702

**Published:** 2026-07-22

**Authors:** Yi Yao, Jingping Li, Bo Zhang, Tingting Li, Enqiang Linghu, Xin Li, Ming-chun Zhao

**Affiliations:** 1 Department of Gastroenterology, First Medical Center, Chinese People's Liberation Army (PLA) General Hospital, Beijing, China; 2 Department of Gastroenterology, Second Medical Center, Chinese People's Liberation Army (PLA) General Hospital, Beijing, China; 3 School of Materials Science and Engineering, Central South University, Changsha, China

**Keywords:** aging, immunity, integrative multi-omics, irritable bowel syndrome, machine learning

## Abstract

**Background:**

Irritable bowel syndrome (IBS) is a prevalent functional gastrointestinal disorder with an elusive pathophysiology. Although immune dysregulation and mild mucosal inflammation are recognized as important factors in IBS, the specific contribution of immunosenescence remains unclear. Prior MR studies on IBS focused on single molecular traits; however, an integrated multi−omics framework for aging−immune genes has not been applied. Here, we integrate cis−eQTL/pQTL/mQTL with machine learning and *in vivo* validation to identify putatively causal biomarkers and unravel the aging−immune axis.

**Methods:**

Aging- and immune-related genes were curated from published databases and analyzed using genome-wide cis-expression quantitative trait loci (cis-eQTL), cis-protein quantitative trait loci (cis-pQTL), and cis-methylation quantitative trait loci (cis-mQTL) datasets. All QTL data were derived from blood or plasma samples. Candidate genes were identified through integrative multi-omics analysis, followed by functional annotation, machine learning-based feature selection, immune infiltration profiling, and drug-target prediction. The expression of key biomarkers was validated using reverse transcription quantitative polymerase chain reaction (RT-qPCR) in colonic tissues from a rat model of diarrhea-predominant IBS (IBS-D).

**Results:**

Integrative multi-omics analysis initially identified 34 high-confidence candidate genes. Through multi-algorithm machine learning such as least absolute shrinkage and selection operator (LASSO), support vector machine recursive feature elimination (SVM-RFE), and random forest, these candidates were refined to a core panel of five biomarkers: the antioxidant enzyme catalase (CAT), acyl-CoA dehydrogenase very long chain (ACADVL), chemokine (C-C motif) ligand 4 (CCL4), cyclin E1 (CCNE1), and Jagged canonical Notch ligand 1 (JAG1). These biomarkers demonstrated strong diagnostic performance for CAT, CCNE1, and JAG1 in the validation cohort (AUC: 0.935–0.951), while ACADVL and CCL4 showed only moderate diagnostic potential (AUC: 0.645–0.649). The combined AUC range in the training cohort was 0.898–0.959. However, in the IBS-D rat model, only JAG1 was significantly upregulated in colonic tissue, whereas CAT, ACADVL, CCL4, and CCNE1 showed no significant changes. Among the five markers, only JAG1 was significantly upregulated in the IBS-D rat colon, confirming its local gut relevance. CAT and CCNE1 showed strong diagnostic performance (AUC >0.9), whereas ACADVL and CCL4 performed modestly (AUC <0.7). Given the blood-derived QTL data and colonic validation, cross-tissue heterogeneity is a key caveat: JAG1 is robustly validated in colon, while the remaining four markers—especially ACADVL and CCL4—require further evaluation in blood, PBMCs, or other relevant tissues. Functional enrichment analysis revealed their involvement in critical biological processes, including oxidative stress, fatty acid metabolism, immune cell recruitment, cell cycle progression, and epithelial-immune crosstalk via Notch signaling. Immune infiltration analysis uncovered a distinct immune landscape in IBS, with significant correlations between biomarker expression and immune cell populations. Notably, only JAG1 was significantly upregulated in the colonic tissues of the IBS-D rat model, confirming its relevance to gut pathophysiology. No significant changes were detected for CAT, ACADVL, CCL4, or CCNE1 in the same tissue samples, highlighting the tissue-specific nature of these candidate biomarkers. The SMR analysis demonstrated consistent causal directions between genetically predicted expression and disease risk for these five markers, with no discordance relative to their upregulation in IBS patients. All biomarker validation was performed at the transcriptomic level (RT−qPCR); no protein−level assays (e.g., Western blot, IHC, or ELISA) were conducted.

**Conclusions:**

This study delineates the genetic architecture linking aging and immunity to IBS through an integrative multi−omics and machine learning approach, providing novel putatively causal evidence and identifying JAG1 as a robustly validated biomarker with diagnostic and therapeutic potential. The remaining candidates warrant further investigation in appropriate biological contexts.

## 1 Introduction

Irritable bowel syndrome (IBS) is one of the most prevalent functional gastrointestinal disorders worldwide, affecting 10-15% of the general population, which is characterized by a constellation of chronic symptoms including recurrent abdominal pain, bloating, and altered bowel habits, in the absence of detectable structural or biochemical abnormalities (1). The presentation of IBS is highly heterogeneous, leading to its sub-classification into constipation-predominant (IBS-C), diarrhea-predominant (IBS-D), mixed (IBS-M), and unsubtyped (IBS-U) forms. This heterogeneity, coupled with a chronic, relapsing-remitting course, profoundly diminishes patients’ quality of life and imposes a substantial economic burden on healthcare systems. Current management strategies are predominantly symptomatic, relying on dietary modifications (e.g., low-FODMAP diets), pharmacological agents such as antispasmodics and antidiarrheals, and psychological therapies (2). However, the efficacy of these interventions is often limited and variable, underscoring an urgent, unmet need for a deeper understanding of the underlying pathophysiology to inform the development of targeted diagnostics and therapeutics.

The pathogenesis of IBS is recognized as multifactorial, involving a complex interplay between the gut-brain axis, visceral hypersensitivity, altered gut motility, intestinal barrier dysfunction, dysbiosis of the gut microbiota, and low-grade immune activation (3). Among these, the role of the immune system has garnered increasing attention (4). A growing body of evidence indicates that a significant subset of IBS patients, particularly those with a post-infectious onset, exhibit subtle but persistent mucosal inflammation (5).Histopathological studies have consistently reported increased infiltration of immune cells, such as mast cells, eosinophils, and T lymphocytes, in the intestinal mucosa of IBS patients (6). These cells release a plethora of pro-inflammatory cytokines (e.g., TNF-α, IL-1β, IL-6) and chemokines, which can sensitize enteric nerves, disrupt epithelial barrier integrity, and dysregulate gut motility, thereby contributing to core IBS symptoms like abdominal pain and altered bowel habits (7–9). This state of chronic, low-grade inflammation suggests a breakdown in immune homeostasis, yet the fundamental triggers and molecular drivers of this immune dysregulation remain incompletely elucidated.

Although immune dysfunction is one of the main focuses in irritable bowel syndrome research, cell senescence may also play an important role in chronic inflammation (10–12) and gut microbiota imbalance (13, 14). However, this relationship is still not explored in IBS research. A polysaccharide/Ussing chamber study (15) showed that increased small intestinal permeability was only significant in elderly patients with diarrhea-predominant IBS (IBS-D), suggesting that age and IBS are ‘symptom dependent’ rather than ‘age dependent’. However, this study did not analyze senescence markers, senescence-associated secretory phenotype (SASP), or underlying mechanisms, leaving the role of cellular senescence in IBS unresolved. Cellular senescence is a state of irreversible growth arrest triggered by various forms of cellular stress, characterized not only by a loss of proliferative capacity but also by a profound secretory phenotype known as SASP. The SASP (16, 17) comprises a diverse mixture of pro-inflammatory cytokines, chemokines, growth factors, and matrix metalloproteinases that can profoundly alter the tissue microenvironment and perpetuate local and systemic inflammation. The concept of inflammaging (18), a chronic, low-grade inflammation that accompanies advanced age, highlights the intricate link between aging and immune decline (immune senescence). While IBS is more prevalent in younger and middle-aged adults, the shared molecular signatures of chronic stress, oxidative damage, and inflammation suggest that accelerated cellular aging processes might be relevant to its pathophysiology, even in non-elderly populations. The potential crosstalk between senescent cells and the immune landscape in the gut, however, has been largely unexplored in the context of IBS.

Traditional observational studies face challenges in establishing causality due to confounding factors and reverse causation. To address this, we employed an integrative multi-omics approach, combining genetic regulatory data(cis-eQTL, cis-pQTL, cis-mQTL) with large-scale IBS transcriptomic and genomic datasets. While individual MR studies have examined specific genetic variants or single molecular traits in relation to IBS, our approach uniquely integrates three QTL layers, machine−learning−based feature selection, and *in vivo* validation in an IBS−D rat model, thereby providing a more comprehensive view of putatively causal pathways. We hypothesized that aging- and immune- related genes contribute to IBS susceptibility through dysregulated expression and protein function. To test this, we integrated multi-omics data to identify candidate genes, refined them using machine learning, and validated key biomarkers in an IBS-D rat model, aiming to uncover genetic links and identify novel targets.

## 2 Materials and methods

### 2.1 Data sources

Aging-related genes were compiled from two sources: 1, 153 genes from published literature and 307 genes from the Human Ageing Genomic Resources (HAGR) database (http://genomics.senescence.info/genes/). An additional 2, 483 immune-related genes were obtained from the ImmPort database (https://www.immport.org/resources). After merging and removing duplicates, a final set of 2, 732 aging- and immune-related genes was curated for downstream analyses.

To investigate genetic regulation of these genes, eQTL (specifically cis-eQTL), cis-pQTL, and cis-mQTL data were acquired. All QTL datasets were derived from blood or blood-derived components, as specified below. Cis-eQTL data were retrieved from the eQTLGen Consortium (https://www.eqtlgen.org/cis-eqtls.html), including variants located within ±1, 000 kb of gene coding sequences (cis) with a significance threshold of p<5×10^–8^. Derived from peripheral blood samples of 31, 684 individuals, this dataset comprised single nucleotide polymorphism (SNP) information for 16, 989 genes. Cis-pQTL data for 4, 895 proteins were obtained from the deCODE database (https://www.decode.com/summarydata/), based on plasma samples of 35, 559 individuals of European ancestry, filtered at p<5×10^–8^. Cis-mQTL data were sourced from the Yanglab (https://yanglab.westlake.edu.cn/software/smr/#DataResource), derived from whole blood samples from the Brisbane Systems Genetics Study (n=614) and the Lothian Birth Cohort (n=1366).

The GWAS summary statistics of IBS (accession GCST90016564) were downloaded from the GWAS Catalog database (https://www.ebi.ac.uk/gwas/studies/GCST90016564), including 53, 400 cases and 433, 201 controls. Transcriptomic data for IBS were acquired from the Gene Expression Omnibus (GEO) database (https://www.ncbi.nlm.nih.gov/gds/) and ArrayExpress database (https://www.ebi.ac.uk/biostudies/arrayexpress), including GSE36701 and E−TABM−176. The GSE36701 dataset served as the training set and initially contained 77 healthy controls, 87 IBS patients, 43 post-Campylobacter infection IBS samples, and 14 Campylobacter-infected non-IBS samples. To minimize batch effects, PCA was performed on all samples and revealed clear separation of batch 1 from other batches, indicating substantial technical variability. Thus, only batch 1 controls (n=40) and all 87 IBS patients (from batch 1) were retained for the training set to ensure internal consistency. We acknowledge potential selection bias, as individual−level covariates were unavailable for formal comparison between retained and excluded controls. However, PCA of global expression distributions between retained batch 1 controls and a random subset of controls from other batches showed no systematic divergence (data not shown). To further mitigate bias, we validated our findings in an independent cohort (E−TABM−176). The validation set E-TABM-176 (ArrayExpress accession) included microarray profiles from sigmoid colon mucosal biopsies of 55 healthy controls and 76 IBS patients (with reported subtype distribution covering IBS−D, IBS−C, and IBS−M, as annotated in the original study). We downloaded the raw “.CEL” files, processed the data using the affy package (V: 1.74.0, https://bioconductor.org/packages/release/bioc/html/affy.html), and normalized the data using the RMA algorithm. For both GSE36701 and E-TABM-176 datasets, we annotated probes using the GPL570 platform annotation file. When multiple probes corresponded to the same gene, the average value was taken as the gene expression level. In subsequent analyses, GSE36701 was used as the training set and E-TABM-176 as the validation set. All human transcriptomic datasets were de−identified and used in accordance with the data access and usage terms specified by the respective repositories (GEO and ArrayExpress).

### 2.2 Summary-data-based Mendelian randomization

The SMR method extends the MR framework to test for pleiotropic associations between genetically influenced molecular traits (e.g., gene expression, DNA methylation, or protein levels) and complex outcomes like disease phenotypes. Accordingly, the SMR software (Linux version 1.3.1) was obtained from the official website (https://yanglab.westlake.edu.cn/software/smr) and applied using default parameters with the following thresholds: FDR-adjusted *p*SMR<0.05 and *p*HEIDI>0.05. HEIDI (p > 0.05) supports pleiotropy over LD-driven linkage, confirming genuine association. Multiple testing was corrected by Benjamini–Hochberg FDR. Genes demonstrating a significant causal association with IBS based on this analysis were selected as candidate genes for subsequent investigation.

### 2.3 Functional annotation of candidate genes

The chromosomal distribution of the candidate genes was visualized using the Rcircos package (v1.2.2) to generate a circular ideogram. To investigate their biological functions and associated pathways, Gene Ontology (GO) and Kyoto Encyclopedia of Genes and Genomes (KEGG) enrichment analyses were performed with the clusterProfiler package (v4.7.1.3), applying a significance threshold of P < 0.05. Furthermore, a protein-protein interaction (PPI) network was constructed by querying the candidate genes against the STRING database (https://string-db.org) and visualized using Cytoscape software (v3.6.0) to summarize potential functional interactions.

### 2.4 Differential expression analysis and machine learning

Differential expression analysis between IBS and control samples in the training set was conducted using the limma package (v3.52.4) to identify differentially expressed genes (DEGs) under a false discovery rate (FDR) threshold of < 0.05. To refine biomarker selection, three machine learning algorithms were employed: Features were z-score normalized before machine learning, especially critical for linear-kernel SVM. Least absolute shrinkage and selection operator (LASSO) logistic regression via the glmnet package (v4.1.7) with 10−fold cross−validation. The optimal λ was selected via the 1−SE rule from a 100−value sequence to balance sparsity and performance. Support vector machine recursive feature elimination (SVM-RFE) implemented with the caret package (v6.0.94) using a linear kernel, 10−fold cross−validation, and a stopping criterion of minimum feature number = 2. C was evaluated at 0.1, 1, and 10; C = 1 was selected for lowest cross−validation error. Random forest (RF) -based feature selection using the randomForest package (v4.7.1.1) with ntree = 500 and mtry = √p (where p is the number of features). Minimum node size = 1, unrestricted depth (default), and OOB error monitoring. The final biomarker panel was defined as the intersection of feature genes selected by all three algorithms. The diagnostic performance of these biomarkers was assessed by generating receiver operating characteristic (ROC) curves and calculating the area under the curve (AUC) with the pROC package (v1.18.4). Their expression levels were compared between IBS patients and controls, and differential expression along with predictive reliability were further validated in an independent cohort.

### 2.5 Gene Set enrichment analysis

Following biomarker identification, Gene Set Enrichment Analysis (GSEA) was performed on the training dataset to elucidate their associated biological pathways. We replaced the median−based stratification with a continuous−phenotype pre−ranked GSEA approach. For each biomarker, we fitted a linear model using limma, with biomarker expression as a continuous predictor, and ranked all genes by the signed moderated t−statistic. Pre−ranked GSEA was then performed with 10, 000 phenotype permutations and Benjamini–Hochberg correction; pathways with FDR < 0.05 were considered significantly enriched.

### 2.6 Immune infiltration analysis

To characterize the immune microenvironment in IBS, single-sample gene set enrichment analysis (ssGSEA) was implemented using the GSVA package (v1.42.0) to calculate enrichment scores for 28 immune cell types in the training set only. This analysis was not replicated in E−TABM−176 because of cross−platform and processing heterogeneity; hence, findings are exploratory and need independent validation with harmonized datasets. Gene signatures defining each immune cell population were derived from a published study. Enrichment scores were compared between IBS patients and controls, with results visualized using heatmaps and box plots. Spearman correlation analysis between immune cell enrichment scores and biomarker expression levels was performed using the psych package (v2.1.6).

### 2.7 Drug-target prediction

To identify potential therapeutic agents for IBS, the candidate biomarkers were queried against the DGIdb database (https://www.dgidb.org) to predict drug-gene interactions. Subsequently, the resulting interaction network was visualized using Cytoscape software.

### 2.8 IBS-D model construction

Animals and ethics. All animal experiments were approved by the Experimental Animal Ethics Committee of Chinese PLA General Hospital (Approval No. SQ2021314).and conducted in accordance with the National Institutes of Health Guide for the Care and Use of Laboratory Animals (8th edition, 2011). Following a one-week acclimatization period, twelve female specific pathogen-free (SPF) Sprague–Dawley rats (160 ± 10 g) were randomly allocated to either a control group or an IBS-D model group (n = 6 per group). Female rats were selected because IBS is 2–3 times more prevalent in women, and female sex hormones critically regulate gut motility, visceral sensitivity, and stress responses—core pathophysiological features of IBS−D. However, we acknowledge that this single−sex design precludes assessing sex−specific effects, as discussed later. The IBS-D model was established using a heterotypic chronic-acute stress paradigm over three consecutive weeks, followed by one week of rest. This protocol incorporated various stressors including cold exposure, water or food deprivation, horizontal oscillation, tail clamping, continuous illumination, and heat exposure. During the final week, animals additionally received daily restraint stress to simulate psychological stress. Control rats were maintained under standard conditions without exposure to any stress induction.

### 2.9 Behavioral and physiological assessments

#### 2.9.1 Body weight and sucrose intake

Body weight and consumption of 1% sucrose solution were recorded on days 0, 7, 14, 21, and 28. For sucrose preference testing, animals were first given free access to 1% sucrose solution for 48 hours, followed by 24 hours of water deprivation. Sucrose intake was then measured during a 1-hour test period.

#### 2.9.2 Abdominal withdrawal reflex test

Visceral sensitivity was evaluated using the AWR test. Following a 12-hour fast, a lubricated air-inflatable balloon was inserted 6 cm into the rectum under light ether anesthesia. After a 20-minute adaptation period, graded colonic distension pressures (0–35 mmHg in 5-mmHg increments) were applied, each lasting 20 seconds with 1-minute intervals. AWR scores were recorded using a standardized scale (2: mild abdominal contraction; 4: body arching and pelvic lifting). The visceral pain pressure threshold was assessed on days 0, 7, 14, 21, and 28.

#### 2.9.3 Defecation quantification via water avoidance stress

Stress-induced defecation was evaluated using the water avoidance stress (WAS) paradigm. Rats were individually placed on a raised platform (10 × 8 × 8 cm) in a water-filled container for 1 hour, with the water level maintained 1 cm below the platform surface. Fecal output (including both formed and soft pellets) and diarrheal episodes were recorded daily over five consecutive days (days 28–32), and the average values were used for statistical analysis. The number of fecal pellets (hard and soft) or diarrhea frequency was recorded across 5 consecutive days (days 28–32), and the average was used for analysis.

### 2.10 RT-qPCR analysis

Total RNA was extracted from tissues using TRIzol reagent (Invitrogen). Complementary DNA (cDNA) was synthesized from total RNA using M-MLV Reverse Transcriptase with random primers (Promega, Madison, WI, USA) according to the manufacturer’s protocol. RT-qPCR was subsequently performed on a ViiA™ 7 Real-Time PCR System (Applied Biosystems, Grand Island, NY, USA) using SYBR Green PCR Master Mix (Applied Biosystems, Thermo Fisher Scientific, Waltham, MA, USA). GAPDH was used as the internal reference gene for normalization. Primer sequences are listed in **Table 1**. Our validation is limited to mRNA expression; protein levels were not assessed via Western blot, IHC, or ELISA.

**Table 1:** The primers information of genes.

Name		Primer sequence (5’to3’)	Primer size(bp)
CCL4	Forward	CCACTTCCTGCTGCTTC	76
CCL4	Reverse	CTGCTGGTCTCATAGTAATCC	
CCNE1	Forward	TGATTCAGCGTGCGTGGAC	134
CCNE1	Reverse	AGACGGGAAGTGGGGAGG	
ACADVL	Forward	CAAAGGAAAGGAACTCACG	162
ACADVL	Reverse	CCACTGCGACTCAACTCT	
CAT	Forward	ATTGCCGTCCGATTCTC	108
CAT	Reverse	GTCCCAGTTACCATCTTCAGT	
JAG1	Forward	CACTACTATGGCTTTGGC	185
JAG1	Reverse	CAGTCACCTGGGAGTTT	
GAPDH	Forward	CGTATCGGACGCCTGGTT	83
GAPDH	Reverse	AGGTCAATGAAGGGGTCGTT	

### 2.11 Histopathological examination

On day 33, all rats were euthanized. For euthanasia, rats were anesthetized by intraperitoneal injection of 5% pentobarbital sodium solution (40 mg/kg) and then exsanguinated via the abdominal aorta. Death was confirmed before tissue collection. After euthanasia, segments of the distal colon (2–4 cm from the anal verge) were collected for histopathological assessment. The tissue specimens were fixed in 4% paraformaldehyde, embedded in paraffin, and sectioned at 4 μm thickness using a manual rotary microtome (HistoCore BIOCUT; Leica Biosystems, Shanghai, China). Finally, the sections were stained with hematoxylin and eosin (H&E) for microscopic morphological evaluation.

### 2.12 Statistical analysis

All statistical analyses were performed using R software (v4.1.2). Group differences were assessed using the Wilcoxon rank-sum test for two-group comparisons and the Kruskal–Wallis test for multi-group comparisons. For exploratory analyses, no adjustment for multiple comparisons was applied, and *P* < 0.05 was considered statistically significant. Post−hoc power analyses based on RT−qPCR effect sizes confirmed adequate power (>0.85, two−sided t−test, α=0.05, n=6/group) for JAG1 (*p*<0.001), but insufficient power to detect small−to−moderate effects for the other four genes, whose non−significant results should be interpreted cautiously.

## 3 Results

### 3.1 Identification of high-confidence candidate genes

**Figure 1:**
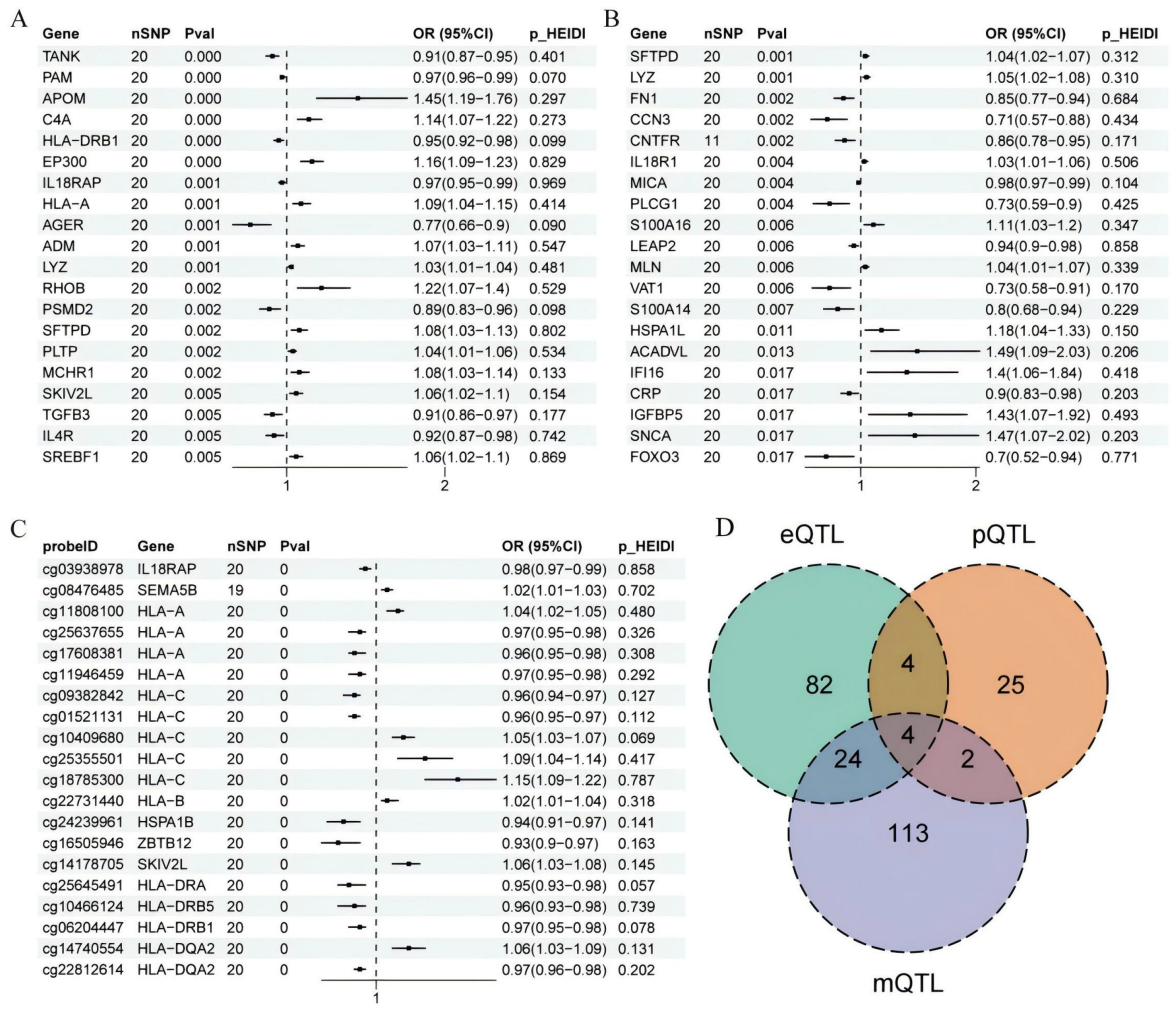
Integrative multi-omics analysis of aging-immune genes and IBS. **(A)** Forest plot of cis-eQTL association with IBS, **(B)** Forest plot of cis-pQTL association with IBS, **(C)** Forest plot of cis-mQTL associations with IBS, **(D)** Venn diagram showing the intersection of candidate genes identified by eQTL, pQTL, and mQTL analyses.

Integrative multi-omics analysis (combining cis-eQTL, cis-pQTL, and cis-mQTL and IBS GWAS datasets) initially identified 114 genes, 35 proteins, and 143 methylation sites potentially causal for IBS (**Figures 1A–C**). All identified signals met the SMR significance (FDR−adjusted pSMR < 0.05) and HEIDI (pHEIDI > 0.05) criteria, confirming they were not LD−driven. To ensure robustness, only signals supported by at least two molecular QTL types were retained, resulting in 34 high-confidence candidate genes (**Figure 1D**).

### 3.2 Functional characterization of candidate genes

**Figure 2:**
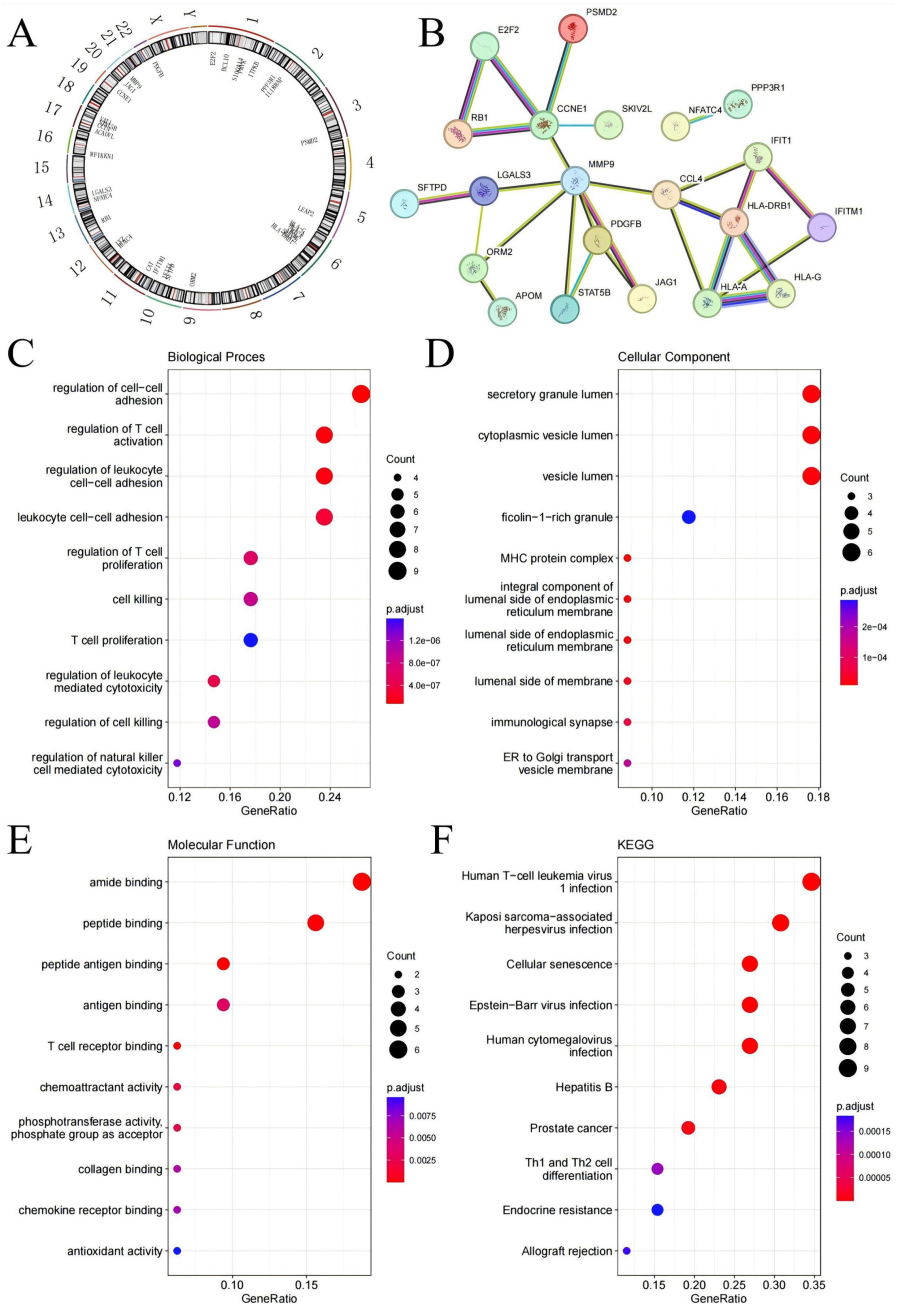
Functional analysis of candidate genes. **(A)** Circular ideogram showing chromosomal distribution of 34 candidate genes. **(B)** Protein Protein interaction (PPI) network diagram for 34 candidate genes. **(C–E)** The Gene Ontology (GO) analysis for GSEA of candidate genes.C: Biological Process;D: Cellular Component; E: Molecular Function. **(F)** The Kyoto Encyclopedia of Genes and Genomes (KEGG) analysis for GSEA of candidate genes.

The 34 candidate genes were distributed across multiple chromosomes (**Figure 2A**). The PPI network revealed hub genes (e.g., MMP9, CCL4) with high connectivity, indicating their potential biological importance (**Figure 2B**). GO enrichment analysis identified 574 biological process (BP) terms (e.g., regulation of cell-cell adhesion), 58 cellular component (CC) terms (e.g., secretory granule lumen), and 49 molecular function (MF) terms (e.g., amide binding)(**Figures 2C–E**). KEGG pathway analysis showed enrichment in human T-cell leukemia virus 1 infection, Th1 and Th2 cell differentiation, and endocrine resistance (**Figure 2F**).

### 3.3 Machine learning-based biomarker selection

**Figure 3:**
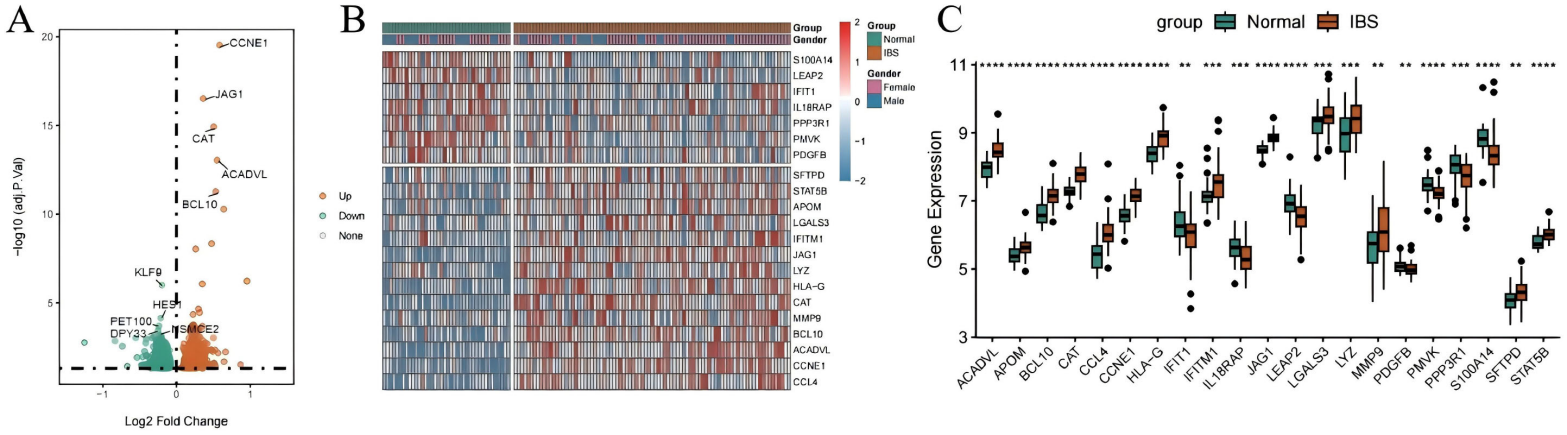
Identification of differentially expressed candidate genes (DEGs). **(A)** Volcano plot illustrating DEGs between controls and IBS samples in the GSE36701 dataset (red: upregulated, blue: downregulated). **(B)** Heatmap of 21 differentially expressed candidate genes. **(C)** Box plots representing the expression profiles of these 21 candidate genes in IBS and control samples (**p < 0.01; ****p<0.0001).

**Figure 4:**
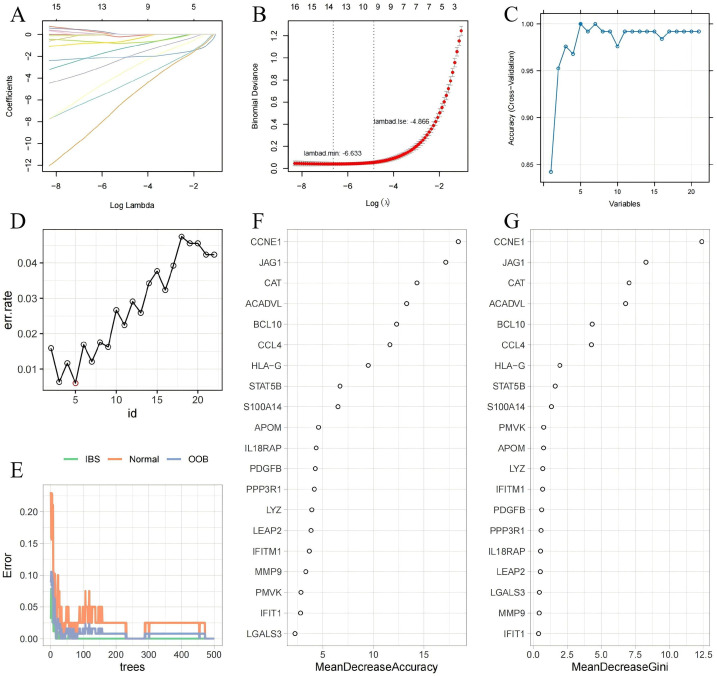
Machine learning-based feature selection. **(A, B)** LASSO logistic regression algorithm: **(A)** Coefficient path diagram **(B)** Cross-validation curve **(C)** SVM-RFE algorithm, the variation curves of predicted true values and error values for each gene are shown. **(D–G)** Feature importance identification based on the Random Forest algorithm: **(D)** OOB error rate **(E)** Mean decrease accuracy **(F)** Mean decrease Gini **(G)** Variable importance plot.

Differential expression analysis in the training set identified 881 DEGs, comprising 436 upregulated and 445 downregulated genes (**Figures 3A, B**). Comparison with the 34 candidate genes revealed that 21 of them exhibited significant expression alterations in IBS patients compared to controls: 7 were downregulated (e.g., IFIT1, IL18RAP, and LEAP2), and 14 were upregulated (e.g., ACADVL, APOM, and BCL10) (**Figure 3C**). These 21 genes were subsequently subjected to machine learning-based feature selection:

LASSO identified 13 potential biomarkers (**Figures 4A, B**)
SVM-RFE identified 5 potential biomarkers (**Figure 4C**) and
RF algorithms identified 20 potential biomarkers (**Figures 4D–G**).

### 3.4 Identification of CAT, ACADVL, CCL4, CCNE1, and JAG1 as Biomarkers

**Figure 5:**
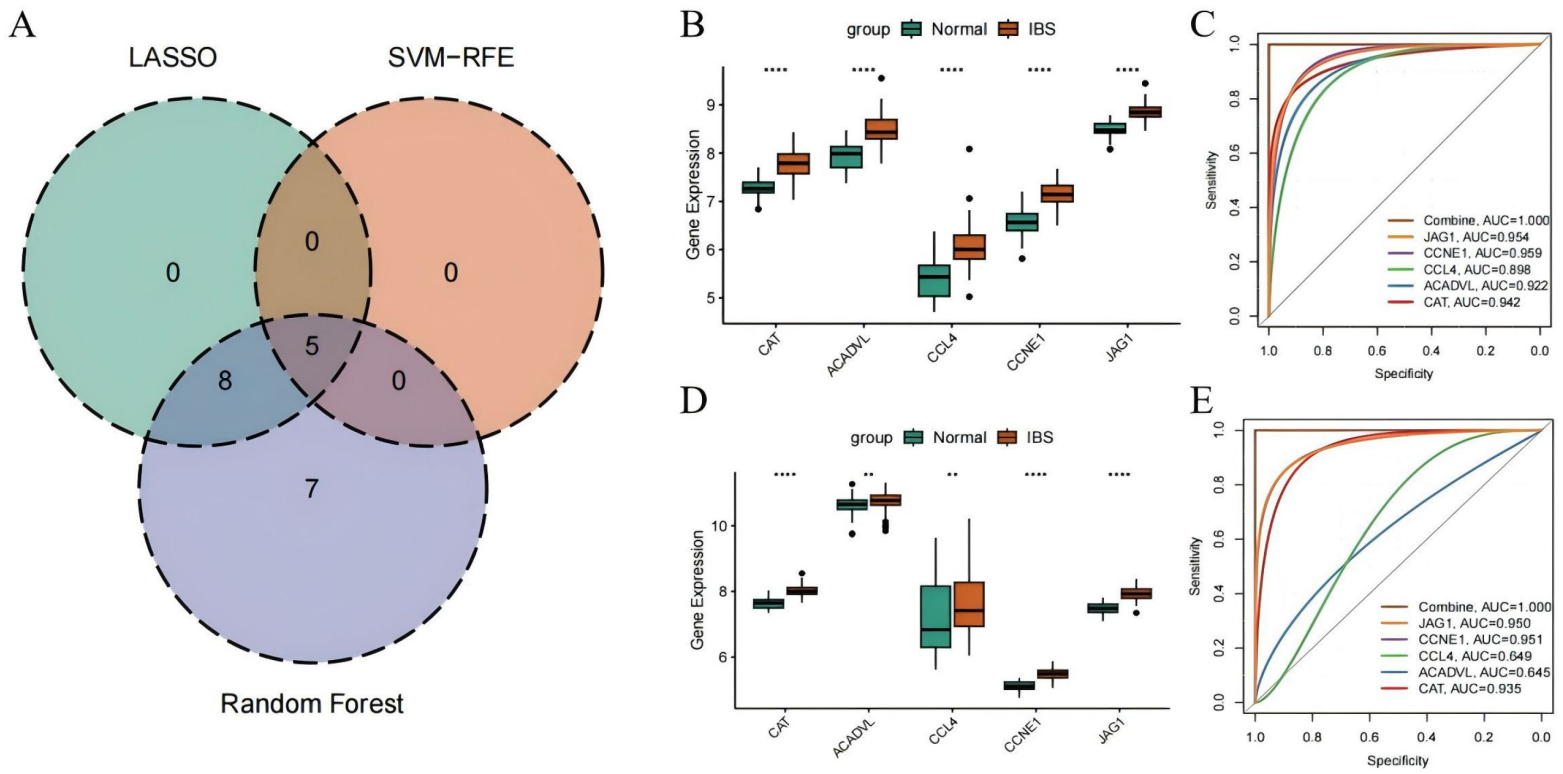
Identification and validation of biomarkers. **(A)** Venn diagram showing the intersection of biomarkers obtained from the three algorithms. **(B)** Expression of the five diagnostic markers in the training set (**p < 0.001). **(C)** ROC curves for the biomarkers in the training set. **(D)** Expression of the five diagnostic markers in the validation set (**p < 0.001). **(E)** ROC curves for the biomarkers in the validation set. **p < 0.01; ****p<0.0001.

The intersection of features identified by the three machine learning algorithms yielded five definitive biomarkers: CAT, ACADVL, CCL4, CCNE1, and JAG1 (**Figure 5A**). Differential expression analysis confirmed significant upregulation of all five biomarkers in both the training (**Figure 5B**) and validation (**Figure 5D**) sets. ROC curve analysis demonstrated strong diagnostic potential for CAT, CCNE1, and JAG1, whereas ACADVL and CCL4 showed moderate performance in the validation set.

Training set: AUC = 0.942 (CAT), 0.922 (ACADVL), 0.898 (CCL4), 0.959 (CCNE1), and 0.954 (JAG1) (**Figure 5C**).

validation set: AUC = 0.935 (CAT), 0.645 (ACADVL), 0.649 (CCL4), 0.951 (CCNE1), and 0.950 (JAG1) (**Figure 5E**).

For all five final biomarkers (CAT, ACADVL, CCL4, CCNE1, and JAG1), the direction of the putative causal effect estimated by SMR (i.e., genetically predicted higher expression increasing IBS risk) was fully concordant with the direction of differential expression observed in both the training and validation cohorts (all upregulated in IBS patients). No discrepancy was identified for these five markers. However, during the initial screening of the 34 candidate genes, a few genes showed opposing directions between SMR causality and case–control expression differences; these were excluded from subsequent machine learning selection to avoid potential confounding or reverse causation.

### 3.5 Functional enrichment analysis of biomarkers

**Figure 6:**
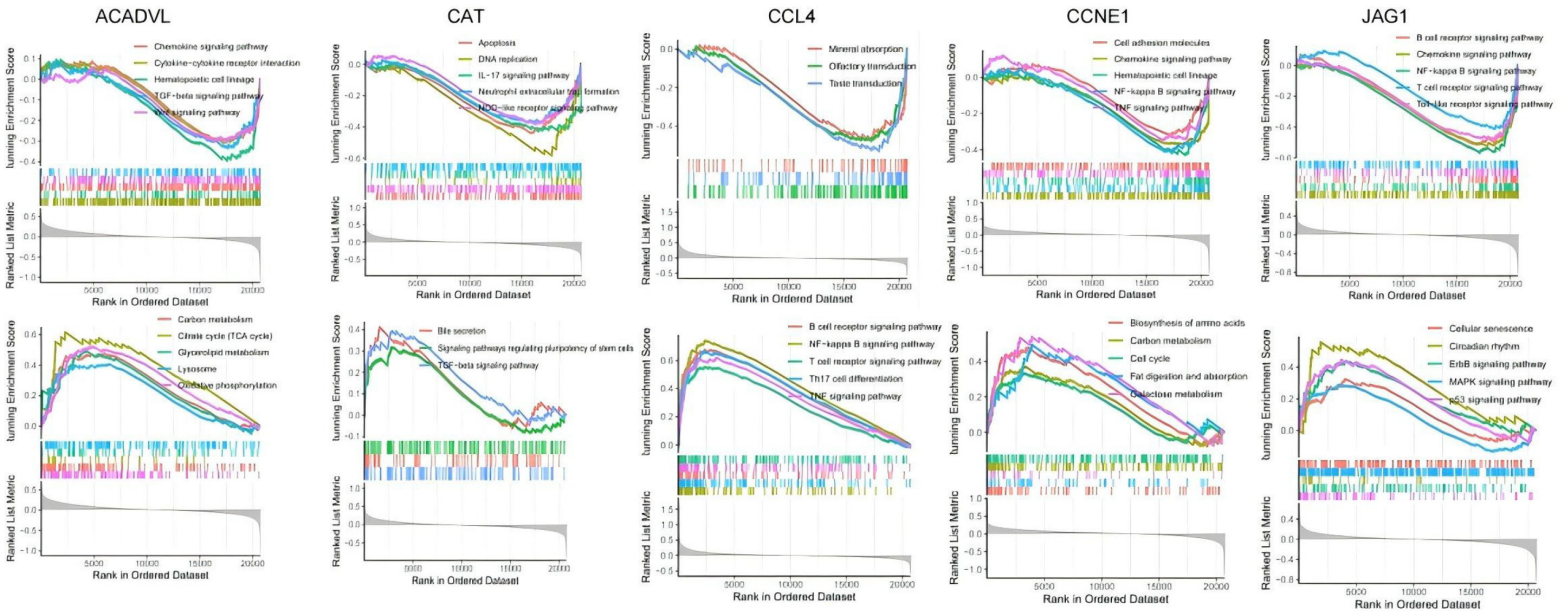
Functional enrichment analysis of biomarkers. Gene Set Enrichment Analysis (GSEA) results for biomarkers (ACADVL, CAT, CCL4, CCNE1, JAG1). The x-axis represents the ranked gene list (by log_2_FC), and the y-axis represents the running enrichment score.

GSEA was performed to elucidate the functional pathways associated with the identified biomarkers (**Figure 6**):

ACADVL: enriched in the TGF-β signaling pathway and carbon metabolism;
CAT: enriched in TGF-β signaling pathway;
CCL4: enriched in mineral absorption and Th17 cell differentiation;
CCNE1: enriched in cell adhesion molecules, the NF-κB signaling pathway, and the TNF signaling pathway;
JAG1: enriched in the B cell receptor and T cell receptor signaling pathways.

The direction and significance of these enrichments remained consistent with our earlier analysis, confirming the robustness of the findings. Notably, the TGF-β signaling pathway showed lower enrichment scores in the ACADVL low-expression group and higher enrichment scores in the CAT high-expression group. The NF-κB signaling pathway showed positive enrichment association with the CCL4 high-expression group, but negative enrichment association with the low-expression groups of both CCNE1 and JAG1.

### 3.6 Immune infiltration analysis and drug prediction for biomarkers

**Figure 7:**
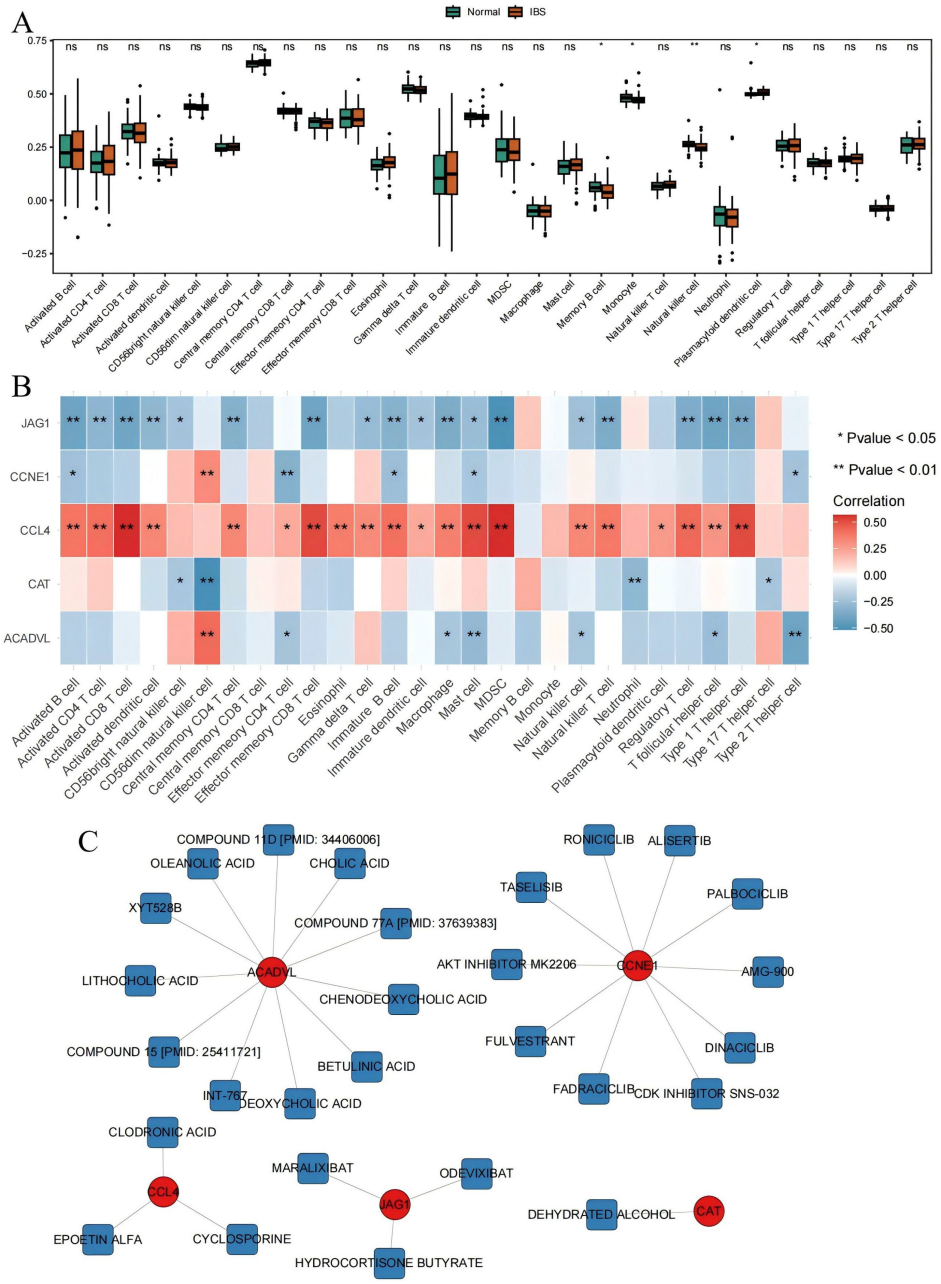
Immune infiltration analysis and drug prediction. **(A)** Box plot visualizing the differences in immune cell infiltration abundance between IBS and control samples for 28 immune cell types. **(B)** Heatmap showing the correlation of immune cell with biomarkers. **(C)** Drug-gene interactions (nodes represent genes/compounds, edges represent interactions). ns p > 0.05; * p < 0.05; **p < 0.01.

In the training cohort, ssGSEA identified four immune cell types with significant differences between IBS and control samples: memory B cells, monocytes, natural killer cells, and plasmacytoid dendritic cells (**Figure 7A**). Correlation analysis showed that CCL4 expression was positively correlated with most immune cells, while JAG1 expression was negatively correlated (**Figure 7B**). Drug-target prediction revealed that ACADVL and CCNE1 interacted with the most compounds (e.g., betulinic acid, roniciclib, cyclosporine) (**Figure 7C**). However, these predictions are solely in silico (DGIdb−derived) and experimentally untested; they serve only as hypothesis−generating clues for future investigation, not as validated therapeutics.

### 3.7 Biomarker validation in the IBS-D rat model

**Figure 8:**
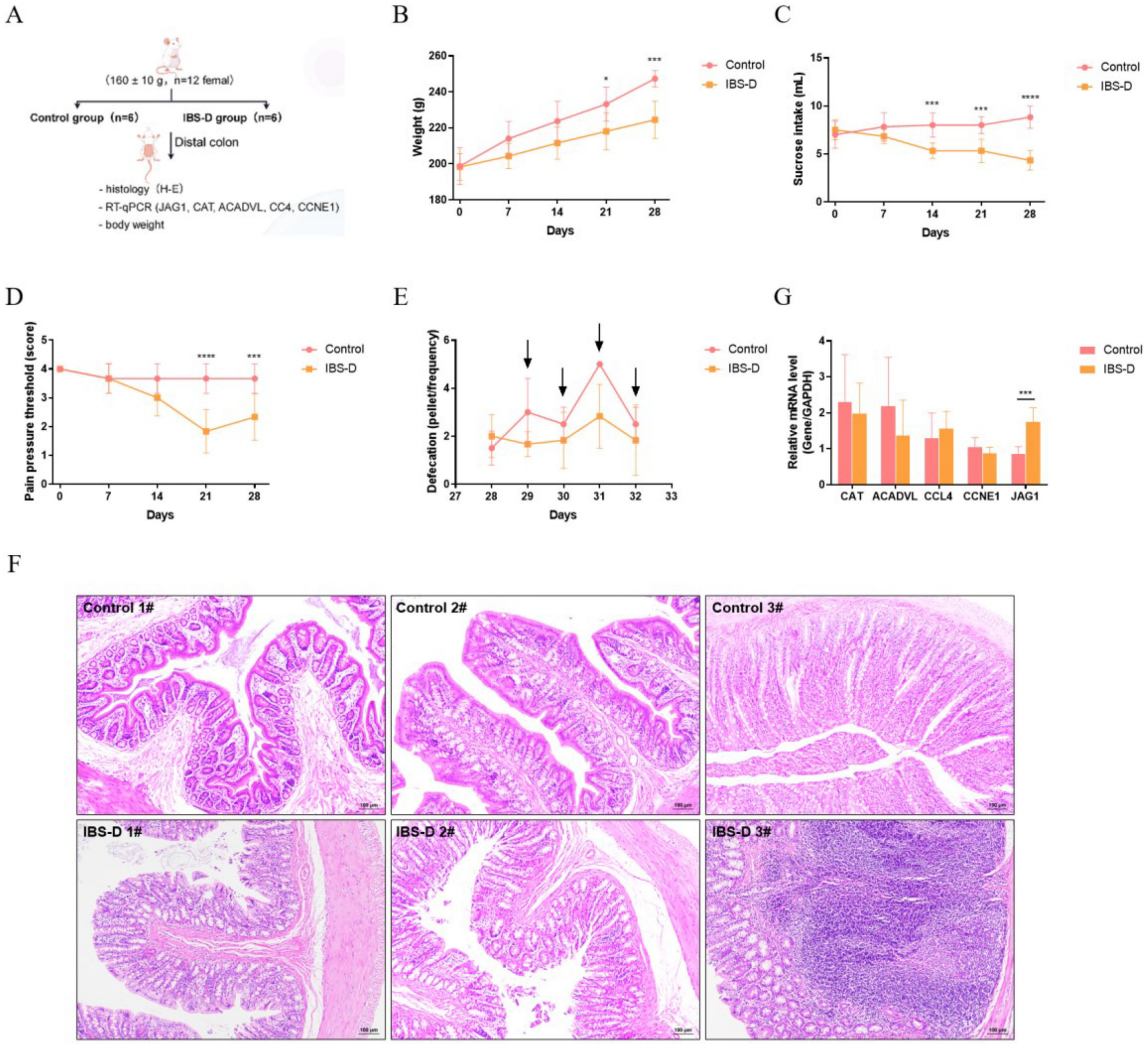
Validation of candidate biomarker in the IBS-D rat colonic tissue. **(A)** Schematic of the IBS-D model construction; **(B)** Body weight changes; **(C)** Sucrose intake; **(D)** AWR scores (**p < 0.01); **(E)** Fecal output and diarrhea episodes (black arrows indicate diarrhea onset); **(F)** H&E staining of colonic tissues (scale bar: 100 μm); **(G)** RT-qPCR analysis of biomarker expression (*p < 0.001). Data are presented as mean ± SD (n = 6 per group). *P < 0.05, ***P < 0.001, ****P < 0.0001.

In the established IBS-D rat model (**Figure 8A**), chronic heterotypic stress exposure induced characteristic pathophysiological changes. Model animals exhibited significantly lower body weight gain and reduced sucrose preference compared to controls, indicating an anhedonic and stress-sensitive phenotype (**Figures 8B, C**). The AWR test demonstrated a significantly lower pain threshold to graded colorectal distension in IBS-D rats (*p* < 0.01, **Figure 8D**), confirming visceral hypersensitivity. During the water avoidance stress period, the IBS-D group developed persistent diarrhea from days 28 to 32 (**Figure 8E**), further supporting successful modeling of diarrhea-predominant IBS.

Histological analysis of distal colon sections confirmed mild mucosal edema and scattered inflammatory cell infiltration in IBS-D rats, contrasting with the intact mucosal architecture observed in controls (**Figure 8F**). While no overt epithelial damage was detected, these morphological alterations are consistent with low-grade inflammation in IBS. We acknowledge that quantitative intestinal permeability and mucosal cytokine measurements were not performed, limiting comprehensive model characterization.

At the molecular level, quantitative PCR analysis of colonic tissues revealed a significant upregulation of JAG1 mRNA in the IBS-D group versus controls (*p* < 0.001, **Figure 8G**). In contrast, the expression levels of CAT, ACADVL, CCL4, and CCNE1 showed no significant differences between groups (*p*> 0.05). Thus, among the five candidate genes identified through human multi-omics and machine learning, only JAG1 was validated in the colonic mucosa of this IBS-D rat model. This validation is based solely on RT−qPCR mRNA data; no protein−level assays were conducted. Notably, our RT−qPCR validation was limited to colonic tissue and did not include blood or PBMCs, despite all QTL data being blood−derived. Thus, direct experimental evidence linking these genetic regulatory signals to colonic expression changes is currently lacking.

## 4 Discussion

### 4.1 Molecular roles of core biomarkers in ibs pathophysiology

This study identified five core biomarkers (CAT, ACADVL, CCL4, CCNE1, and JAG1) through integrative multi-omics and machine learning, shedding light on the molecular links between aging, immunity, and IBS. These biomarkers exhibit strong diagnostic performance and are involved in key biological processes underlying IBS. The observed upregulation of these biomarkers in IBS patients along with their high diagnostic value demonstrated by AUC values supports their potential involvement in disease pathogenesis. Their enrichment in key immune and inflammatory pathways such as TGF-β, NF-κB, TNF, and T/B cell receptor signaling establishes a molecular connection between cellular aging, immune dysregulation, and the chronic low-grade inflammation characteristic of IBS (19–22).

CAT: As an antioxidant enzyme, CAT plays a critical role in mitigating oxidative stress-an established contributor to epithelial damage and barrier dysfunction in IBS (23).Its association with the Transforming growth factor-β (TGF-β) (24, 25) pathway indicates potential dual functions in both mitigating oxidative injury and regulating mucosal repair processes. The positive enrichment of TGF-β signaling in the CAT high-expression groups implies context-dependent roles, potentially shifting from pro-apoptotic to pro-repair under oxidative stress.

ACADVL: Involved in fatty acid β-oxidation, ACADVL supports the emerging concept of epithelial metabolic reprogramming in functional gastrointestinal disorders (26). Its enrichment in carbon metabolism and TGF-β signaling implies novel interactions between energy homeostasis and inflammatory responses. The concomitant lower enrichment of TGF-β signaling in ACADVL low-expression groups suggests that impaired fatty acid oxidation may disrupt immune regulation in the gut mucosa (27).

CCL4: This chemokine is a key regulator of immune cell recruitment. Its upregulation and positive correlation with immune cells, particularly monocytes and plasmacytoid dendritic cells, suggest its potential role as a regulator of the local immune microenvironment (28). Association with T helper cell 17(Th17)cell differentiation corresponds with the growing recognition of this T-cell subset in mucosal immunity and visceral hypersensitivity (29–31).

CCNE1: A regulator of cell cycle progression, CCNE1 upregulation may reflect compensatory epithelial proliferation in response to chronic inflammation (32, 33). Its association with NF-κB and TNF signaling pathways suggests dual roles in cell cycle control and inflammatory cascades (34, 35).

JAG1: A ligand in the Notch signaling pathway, JAG1 was consistently validated across datasets and the IBS-D rat models. Its enrichment in T/B cell receptor signaling and upregulation in colonic tissues highlight roles in epithelial-immune crosstalk. The concurrent lower enrichment of TGF-β1 signaling in JAG1 low-expression group positions JAG1 at the interface of barrier function and immunity-–two systems dysregulated in IBS (36–39).

### 4.2 immunosenescence and metabolic dysregulation in IBS

#### 4.2.1 immunosenescence and gut homeostasis

Immunosenescence, the aging-related decline in immune function, contributes to IBS pathogenesis by impairing immune surveillance, disrupting the mucosal barrier, and perpetuating inflammation. Studies report a positive correlation between immunosenescence severity and IBS symptom burden, indicating a role in disease progression. Mechanistically, immune aging impairs surveillance, compromises the intestinal mucosal barrier, and dysregulates inflammatory responses, thereby worsening visceral hypersensitivity and motility disturbances (15). Concurrently, increased expression of proinflammatory mediators sustains a chronic low-grade inflammatory state that is tightly linked to abdominal pain. Immune senescence also perturbs the gut microbiota, which amplifies immune dysfunction and establishes a vicious cycle. IBS patients with greater immunosenescence often exhibit more severe abdominal pain, altered bowel habits, and treatment resistance. Thus, interventions that target immunosenescence may offer a novel therapeutic avenue for IBS. These findings extend beyond the local gut microenvironment to inform understanding of brain-gut axis dysregulation in IBS pathophysiology (40). Several biomarkers, particularly those involved in immune signaling such as CCL4 and JAG1, may contribute to neuroimmune interactions underlying visceral hypersensitivity and central pain processing. As a chemokine, CCL4 can directly sensitize primary afferent neurons and modulate neurotransmitter release, potentially facilitating communication between immune cells and sensory nerves within the intestinal wall (41). Gene-expression studies of immune aging likewise confirm upregulation of CCL4 during immunosenescence, which coexists with persistent chronic inflammation (42). The enrichment of JAG1 in T cell receptor signaling aligns with emerging evidence regarding T cell mediation of stress-induced visceral hypersensitivity through effects on enteric nerves (43). Additionally, metabolic alterations indicated by ACADVL dysregulation may influence neuronal energy metabolism, potentially affecting neural function and pain perception (44). Subsequent investigations should examine potential correlations between these peripheral biomarkers and central nervous system changes through neuroimaging or assessment of stress response pathways to better understand their role in integrated brain-gut dysfunction.

#### 4.2.2 Oxidative stress and metabolic alterations

The prominence of CAT and ACADVL within the biomarker panel underscores the underappreciated significance of oxidative stress and metabolic remodeling in IBS. In a mouse immunosenescence model of D-galactose, umbilical cord mesenchymal stem cells (UC-MSCs) treatment could delay the thymus and spleen atrophy, reduce the number of aging-related β-galactosidase-positive cells and lower the expression of aging-related genes p16, p53, p21 and RB at molecular level. In addition, treatment with UC-MSCs increased antioxidant enzymes including CAT, superoxide dismutase (SOD) and glutathione peroxidase (GSH-PX) activities and decreased the level of Malondialdehyde (MDA) levels by activating Nuclear factor erythroid 2-related factor 2/Heme oxygenase-1(Nrf2/HO-1) signal pathway, alleviating oxidative stress and improving immune system function. This mechanism shows that CAT plays a key role in maintaining the antioxidant capacity of immune system (45). CAT upregulation indicates an adaptive response to increased reactive oxygen species production potentially originating from mucosal inflammation, mitochondrial dysfunction, or bacterial metabolites. Beyond its antioxidant capacity, CAT’s association with apoptosis and TGF-β signaling supports its potential involvement in regulating epithelial turnover and mucosal healing (46, 47). Recent multidimensional bioinformatics analyses indicate that ACADVL not only occupies a central role in lipid metabolism but also associates closely with metabolic reprogramming in immune cells (48). While epithelial cells in the stressed gut may undergo metabolic reprogramming resembling the Warburg effect, shifting toward aerobic glycolysis despite oxygen availability. Such metabolic adaptation could influence barrier function, immune responses, and neuronal signaling. The connection between ACADVL and TGF-β signaling further indicates that metabolic alterations might modulate fibrotic responses observed in chronic IBS cases, particularly constipation-predominant subtypes. Future research should systematically characterize the metabolic profile of IBS epithelium and evaluate whether modulation of specific metabolic pathways through dietary interventions or metabolic inhibitors can ameliorate IBS symptoms.

### 4.3 Limitations and methodological considerations

While this work provides novel insights, several limitations must be acknowledged. Future investigations should prioritize larger, well-characterized cohorts to establish the diagnostic utility of these biomarkers in peripheral blood and explore their variation across IBS subtypes. Furthermore, subsequent validation efforts would benefit from standardized pre-analytical processing protocols and longitudinal sampling designs to account for diurnal rhythms and symptom-related fluctuations in biomarker expression. Key limitations include mRNA−only human data (no protein validation), lack of blood/PBMC validation for the four non−JAG1 markers, small animal sample size (n=6, underpowered for moderate effects), and absence of functional perturbation studies—thus causal roles remain inferred, not proven. Furthermore, restricting the training set to a single GSE36701 batch minimized batch effects, improving internal consistency but potentially reducing representativeness and introducing selection bias. Individual−level covariates were unavailable, precluding comparisons between retained and excluded controls. PCA confirmed batch 1 was distinct, justifying this choice. External validation in an independent cohort partially mitigates this concern, as key biomarkers (CAT, CCNE1, JAG1) maintained high AUCs, suggesting the findings are not artefactual. Larger, multi−batch training sets with full clinical annotations are needed to quantify and adjust for selection bias.

A notable finding of this study is the reduced diagnostic performance of ACADVL and CCL4 in the independent validation set (AUC < 0.7) compared to the training set. Several factors may account for this decline. First, differences in cohort demographics, such as age, sex distribution, and disease severity, could influence gene expression levels. Second, IBS is clinically heterogeneous; the validation set may have contained a different distribution of subtypes (e.g., fewer post−infectious or diarrhea−predominant cases), which might affect the expression of immune− and metabolism−related genes. Third, although both datasets used sigmoid colon biopsies, subtle variations in sampling depth or tissue processing cannot be excluded. Fourth, platform differences (Affymetrix U133 Plus 2.0 vs. U133A/B) and normalization methods may introduce systematic biases, particularly for genes with moderate expression levels. Similarly, ssGSEA immune infiltration was performed only in the training set (not in E−TABM−176) because of platform heterogeneity, making the correlations exploratory and awaiting validation with harmonized data. These possibilities highlight the need for future multi−center, well−phenotyped cohort studies to clarify the roles of ACADVL and CCL4 in IBS pathophysiology. While this approach improved internal consistency, it may have reduced sample representativeness and introduced selection bias. The subsequent external validation using an independent cohort partially mitigates this concern, but future studies with larger, multi−batch training sets are warranted. It should be noted that GSEA identifies associations between biomarker expression levels and enrichment of pathway−related genes; it does not provide direct evidence of pathway activation or suppression, which would require targeted functional experiments.

An additional important limitation is the cross−tissue heterogeneity inherent in our integrative multi−omics approach. The eQTL, pQTL, and mQTL datasets were all derived from blood or plasma samples, whereas the transcriptomic training and validation sets were derived from colonic mucosal biopsies, and the GWAS summary statistics were population−based. The SMR method integrates these different data types to infer putatively causal relationships at the genetic level, but this does not guarantee that genes identified via blood−derived QTLs are differentially expressed in the disease−relevant tissue (colon). This heterogeneity may partly explain why ACADVL and CCL4, which showed good performance in blood−derived QTL analyses, exhibited only moderate diagnostic performance in colonic transcriptomic validation and no significant changes in rat colonic tissue. Cross-tissue correlation analyses, though valuable for putative causal inference, were precluded by the absence of matched blood–colon samples in public datasets; future studies should adopt paired multi-tissue sampling to systematically evaluate biomarker concordance across tissues.

When integrating SMR−based causal inference with differential expression analysis, we recognize that a positive putative causal estimate from cis−eQTL/SMR does not necessarily guarantee the same direction of differential expression in case–control comparisons. Discrepancies can arise from tissue−specific regulatory effects (e.g., blood−derived eQTL data versus colonic tissue expression), dynamic disease stage−dependent regulation, feedback inhibition, or post−transcriptional modifications. In the present study, all five final biomarkers exhibited concordant directions between SMR−predicted causality and observed expression changes. Nevertheless, for the small subset of candidate genes that initially displayed directional inconsistency, they were intentionally excluded from further analysis to maintain interpretability. We emphasize that future applications of this integrative pipeline should systematically test for directional consistency using independent cohorts and consider discordant candidates as potentially arising from reverse causation or confounding. Our current data do not allow us to experimentally manipulate each biomarker to establish definitive causality; therefore, the inferred putatively causal relationships should be interpreted as genetic support for association rather than proof of mechanistic direction, especially for CAT, ACADVL, CCL4, and CCNE1, which were not validated at the protein level in colonic tissue (no Western blot, immunohistochemistry, or ELISA was performed). Thus, all evidence here is transcriptomic; protein expression and post−translational modifications were not assessed.

The tissue-specific expression patterns of these biomarkers present a critical limitation. While we selected female rats to reflect the higher prevalence of IBS−D in women and the modulatory roles of female sex hormones on gut function, this design necessarily precludes assessment of sex−specific effects and limits extrapolation to male pathophysiology. Consequently, our findings should be interpreted with caution regarding potential sex differences, and future studies with sex−balanced cohorts are warranted. Post−hoc power analysis confirmed adequate power for the large JAG1 effect (*p* < 0.001, power > 0.85) but limited power for moderate effects for CAT, ACADVL, CCL4, and CCNE1, which showed no significant colonic changes. Thus, among the five candidates, only JAG1 was validated in the colonic mucosa, whereas the other four showed no consistent alteration in this tissue. This discrepancy likely arises from tissue−specific regulatory effects, as our QTL data were derived from blood/plasma, whereas validation was performed in colonic mucosa. Consequently, JAG1 appears most relevant to colonic pathophysiology, while the other four biomarkers may predominantly reflect systemic or immune−cell processes and warrant future validation in blood, PBMCs, or other compartments. These considerations underscore the necessity for multi−tissue validation in future research, incorporating parallel analysis of blood, mucosal biopsies, and potentially fecal samples to comprehensively capture different facets of IBS pathophysiology. A further limitation concerns the IBS-D rat model validation. Although behavioral and histopathological assessments confirmed core IBS-D phenotypes (visceral hypersensitivity, stress-induced diarrhea, and low-grade mucosal inflammation), we did not perform quantitative intestinal permeability assays (e.g., FITC−dextran) or mucosal cytokine profiling (e.g., IL−1β, IL−6, TNF−α). These assays are routinely used to evaluate barrier dysfunction and inflammation in IBS models; their omission renders the model characterization incomplete. Future studies should incorporate them for a more comprehensive phenotypic validation.

In addition to the cross−tissue heterogeneity and sex−related limitations discussed above, the divergent diagnostic performance of the five biomarkers in the validation cohort necessitates a hierarchical reclassification of their evidence levels. CAT, CCNE1, and JAG1 each achieved AUC > 0.9, supporting their designation as high−performance candidate biomarkers, whereas ACADVL and CCL4 yielded AUC < 0.7, indicating only modest diagnostic utility. Despite their initial identification through rigorous multi−omics and machine learning, the moderate validation performance of the latter two suggests greater susceptibility to cohort heterogeneity, IBS subtype distribution, or technical platform differences. We therefore classify CAT, CCNE1, and JAG1 as high−performance candidates, and ACADVL and CCL4 as exploratory candidates that require further validation in larger, well−phenotyped, and subtype−stratified cohorts before their clinical or pathophysiological relevance can be firmly established.

Finally, while this research successfully identifies and correlates biomarkers with IBS through putative causal inference methods and animal model validation, the precise molecular mechanisms underlying their contributions to symptomatology remain undefined. Critical questions persist regarding whether JAG1 upregulation directly promotes visceral hypersensitivity through neuronal sensitization or primarily modulates epithelial barrier function, and whether CCNE1-driven proliferation compromises epithelial integrity. The current data cannot resolve these mechanistic questions, nor can they elucidate the potential interactions between these biomarkers and their collective contribution to disease phenotypes. It remains plausible that these biomarkers operate within interconnected molecular networks rather than functioning in isolation, potentially forming circuits that sustain the chronicity of IBS symptoms, thus representing an important avenue for future investigation.

### 4.4 From biomarkers to therapies: a roadmap for functional validation and translation

#### 4.4.1 Implications for IBS subtyping and personalized medicine

The well-established heterogeneity of irritable bowel syndrome presents persistent challenges in its diagnosis and therapeutic management. The identified biomarker panel, encompassing immune activation markers CCL4 and JAG1, oxidative stress indicator CAT, metabolic mediator ACADVL, and proliferation marker CCNE1, provides a potential molecular framework for stratifying IBS patients into distinct pathophysiological subgroups. This stratification suggests the existence of an immune-inflammatory subtype characterized by elevated expression of immune-related biomarkers, potentially exhibiting different treatment responses compared to subgroups dominated by metabolic or oxidative stress profiles. Such molecular classification aligns with the contemporary understanding of IBS as a spectrum disorder where diverse biological mechanisms converge to produce similar manifestations. Further research should validate whether these biomarkers can identify relevant subgroups with distinct disease trajectories, comorbidity patterns, and treatment outcomes. The integration of molecular signatures with conventional parameters could ultimately facilitate personalized treatment approaches, transitioning from symptom-based management toward mechanism-targeted interventions.

#### 4.4.2 Future directions and translation

Current limitations naturally define future priorities in progressing from biomarker discovery to mechanistic insight and clinical implementation. Functional validation using advanced *in vitro* models, such as intestinal organoids and epithelial cell systems, represents an essential next step. Genetic manipulation via siRNA knockdown or CRISPR−Cas9 can clarify JAG1 roles in epithelial barrier integrity, secretion, and chemokine regulation. Overexpression studies should test whether JAG1 is sufficient to induce visceral hypersensitivity in neuron−epithelial co−cultures, while parallel work should characterize ACADVL’s metabolic functions and impact on inflammation. CCL4’s immunosenescence properties require investigation through neuronal excitability and mast cell activation studies. Previously identified drug interactions involving ACADVL and CCNE1 offer a rational starting point for pharmacological screening.

Clinical translation requires systematically examining relationships between the identified biomarkers and existing IBS therapies. Mapping connections between these biomarkers and known drug targets may reveal novel mechanisms and support biomarker−guided patient stratification. The immune signature associated with CCL4 and JAG1 justifies exploring immunomodulators (e.g., mast cell stabilizers or anti−cytokine therapies) in stratified populations. Meanwhile, CAT’s link to oxidative stress suggests evaluating antioxidants or NRF2 activators in relevant subsets, particularly post−infectious or diarrhea−predominant patients. This leads to personalized strategies: immunomodulators (e.g., anti−CCL4) for immune−inflammatory subtype; antioxidants or metabolic inhibitors for metabolic subtype; and CCNE1 inhibitors (e.g., roniciclib) for proliferative subtype. Predicted drug−target pairs (e.g., betulinic acid for ACADVL, roniciclib for CCNE1, cyclosporine for CCNE1) need preclinical validation, and longitudinal studies should track biomarker dynamics throughout disease course and treatment. All drug−target interactions reported here are purely computational, derived from DGIdb database−mining, and lack any experimental verification in IBS models or human tissues. For compounds such as betulinic acid, roniciclib, and cyclosporine, their actual binding affinity, target specificity, off−target effects, and—critically—therapeutic efficacy and safety in IBS remain entirely unknown. Accordingly, we present these predictions solely as a hypothesis−generating tool to prioritize molecular targets (e.g., ACADVL and CCNE1) for future mechanistic studies, not as validated therapeutic leads. Prior to any *in vivo* testing, rigorous functional validation is essential, including biochemical assays (e.g., surface plasmon resonance, cellular thermal shift) to confirm target engagement, cell−based assays in intestinal epithelial or immune lines to assess phenotypic outcomes, and ex vivo studies using patient−derived biopsies or organoids to verify biological relevance. Only then would animal efficacy studies be justified. We acknowledge this as a major limitation and have revised our interpretation throughout the manuscript to avoid overstatement. However, it is critical to emphasize that CCNE1 is a master regulator of the cell cycle, and systemic inhibition of CCNE1 (e.g., with roniciclib) may cause significant adverse effects, including myelosuppression, gastrointestinal toxicity, and impaired tissue renewal. Therefore, any future exploration of CCNE1 as a therapeutic target in IBS would require highly selective delivery strategies (e.g., gut−restricted or epithelium−specific targeting) to avoid systemic complications. At this stage, these predicted interactions should be viewed strictly as hypothesis−generating, not as clinical recommendations. The emerging link between cellular aging and IBS pathophysiology warrants focused research on senescence−associated secretory phenotypes and their role in chronic inflammation. Assessing senescent cell burden in IBS mucosa and testing senolytic compounds in preclinical models may uncover new therapies. Longitudinal monitoring of biomarker changes throughout disease and treatment will help determine their potential as surrogate endpoints. Ultimately, integrating these molecular signatures with microbiome and metabolomic data through systems biology will advance the understanding of IBS pathogenesis and enable precision medicine approaches.

## 5 Conclusions

This study integrates putative causal inference, machine learning, and experimental validation to unveil the genetic architecture linking aging and immunity to IBS, offering new insights into its pathophysiology and identifying diagnostic biomarkers and potential therapeutic targets. The key conclusions are summarized as follows:

1. This study provides novel genetically supported evidence linking aging− and immune−related genes to IBS susceptibility, extending previous genetic association studies by integrating multi−omics data (eQTL, pQTL, mQTL) with machine learning and experimental validation.
2. A set of 34 high-confidence candidate genes was identified and subsequently refined to five core biomarkers (CAT, ACADVL, CCL4, CCNE1, and JAG1), through multi-algorithm machine learning. Of these, CAT, CCNE1, and JAG1 exhibited strong diagnostic potential for IBS (AUC > 0.9 in validation), while ACADVL and CCL4 showed only moderate diagnostic performance (AUC < 0.7), and are therefore reclassified as preliminary, requiring validation in larger, stratified cohorts and relevant tissues.
3. These biomarkers are functionally implicated in key IBS-related biological processes, including oxidative stress response, fatty acid metabolism, immune cell recruitment and inflammation, cell cycle progression, and Notch signaling that influences epithelial-immune crosstalk.
4. Immune infiltration analysis revealed a distinct immune landscape in IBS and significant correlations between the biomarkers and specific immune cell populations, reinforcing the role of immune dysregulation in IBS pathogenesis.
5. Experimental validation confirmed the significant upregulation of JAG1 in the colon of a well-characterized IBS-D rat model, underscoring its relevance to local gut pathophysiology. No significant changes were found for CAT, ACADVL, CCL4, or CCNE1 in the same samples, indicating that JAG1 is the most robustly validated biomarker from the animal model. Among the remaining four genes, CAT and CCNE1 have strong human diagnostic utility but no rat colonic validation, while ACADVL and CCL4 show only modest human performance and no rat change. We therefore classify ACADVL and CCL4 as exploratory candidates, warranting further validation in larger, sex−balanced cohorts and alternative samples.

Overall, JAG1 holds promise as a diagnostic and therapeutic target for IBS. Given the cross-tissue design, validation in disease-relevant tissue is essential. We acknowledge that our RT−qPCR validation, performed only in colonic tissue without blood/PBMC assays, leaves direct evidence connecting blood−derived QTL signals to colonic expression currently unavailable. JAG1 is confirmed in colonic mucosa, whereas the other four markers require further evaluation in blood, immune cells, or other gut segments to clarify their roles in IBS pathophysiology. The study provides a novel molecular framework suggesting that IBS heterogeneity may be driven by distinct underlying mechanisms, including immune-inflammatory, metabolic, and oxidative stress pathways, paving the way for future pathophysiological subtyping and personalized treatment strategies. Validation was at the mRNA level only; protein assays are warranted to confirm functional relevance. Our findings complement and extend the existing literature on IBS genetics by offering a multi−layered putatively causal inference strategy and preclinical corroboration, rather than claiming a singular “first” discovery. We reiterate, however, that these conclusions are limited by the lack of protein validation in human tissues, blood/PBMC data for non−JAG1 markers, a small animal cohort, and functional experiments—all requiring future investigation.

## Data Availability

The original contributions presented in the study are included in the article/**Supplementary Material**. Further inquiries can be directed to the corresponding author.
